# Transaortic Intra-Aortic Balloon Pump Catheter Insertion through a Separate Saphenous Vein Graft in Patients with Severe Aortoiliac Disease

**DOI:** 10.1155/2014/247803

**Published:** 2014-01-02

**Authors:** Faruk Toktas, Senol Yavuz, Cuneyt Eris, Suleyman Surer

**Affiliations:** Department of Cardiovascular Surgery, Bursa Yuksek Ihtisas Education and Research Hospital, 16330 Bursa, Turkey

## Abstract

*Background*. Intra-aortic balloon pump (IABP) is the most widely used mechanical assist device for hemodynamic support in high risk patients undergoing cardiac surgery. The aim of our study was to confirm whether transaortic route is a suitable alternative to allow IABP insertion in patients with severe aortoiliac diseases. *Methods*. This study included 7 consecutive patients undergoing coronary artery bypass grafting for severe coronary artery disease associated with severe aortoiliac disease. These patients could not be weaned from cardiopulmonary bypass and required the IABP support, which were placed through the ascending aorta. IABP catheter was inserted indirectly through a separate saphenous vein graft anastomosed to the ascending aorta by an end-to-side manner under a partial occluding clamp and advanced to the desired position in the descending thoracic aorta and exteriorly brought into the subcutaneous tissues in the jugulum. *Results*. The procedure was successfully performed in all the patients. The mean duration of IABP support was 54.0 ± 13.4 hours. There were no in-hospital mortality and complications related to transaortic route. IABP removal did not require repeat sternotomy. At postoperative 6th month, multislice CT examination showed thrombotic occlusion at the remnant of the saphenous vein graft. *Conclusions*. This technique is a simple, reliable, and reproducible option in patients with severe aortoiliac disease in whom retrograde femoral route is not possible.

## 1. Introduction

Intra-aortic balloon pump (IABP) is the most frequently used mechanical assist device in high risk cardiac surgery patients. Percutaneous femoral IABP catheter insertion using the Seldinger technique for hemodynamic support is commonly an accepted route in patients with preoperative and perioperative cardiac failure by most cardiovascular surgeons [1, 2]. However, this route is not suitable in patients with severe aortoiliac pathologies including severe occlusive aortoiliac disease and aortic and iliac aneurysmal disease or severe peripheral vascular disease. In these complicated settings, placement of IABP catheter may fail or cause complications due to limb ischemia [3]. The incidence of failure for IABP insertion through the femoral route is ranging from 13% to 21% [4, 5].

In high risk patients whose anatomy rendered the transfemoral approach unsuitable, possible alternative insertion sites of IABP catheter are the ascending aorta, the aortic arch, the common iliac artery (retroperitoneal approach), the subclavian, the axillary, and brachial arteries. The best access site is clearly unknown and depends on the surgeon's experience [3, 5–7, 10–14].

The IABP catheter may be inserted through a synthetic graft anastomosed to the ascending aorta [5, 8, 9, 15] or directly transaortic insertion without grafting [7, 16, 17] in patients with severe aortoiliac occlusive disease that precluded a safe transfemoral approach. The technique of transaortic IABP catheter insertion is being evolved. To our knowledge, there are no publications related to the saphenous vein graft usage for IABP catheter insertion.

Here, we present an alternative technique of intraoperative transaortic insertion of the IABP through the ascending aorta using a separate saphenous vein graft anastomosed to the ascending aorta by an end-to-side manner tunneled to the skin above the manubrium sterni in a series of 7 consecutive patients with severe aortoiliac disease.

## 2. Materials and Methods

### 2.1. Patients

This retrospective study included 7 consecutive patients undergoing coronary artery bypass grafting (CABG) for severe coronary artery disease associated with severe aortoiliac disease between January 2008 and December 2012. These patients could not be weaned from cardiopulmonary bypass (CPB) and required assistance with the IABP, which were placed through the ascending aorta. In all of these patients, traditional transfemoral IABP insertion failed or was contraindicated. Institutional Review Board approved the protocol, and informed consent was obtained from each patient. The clinical details of the patients are given in Table 1.

All of the 7 patients in the series were severely symptomatic in New York Heart Association Class III or IV. Their left ventricular ejection fraction ranged from 25% to 30% (mean, 28.3 ± 1.8%). Six patients had severe aortoiliac occlusive disease and aortic aneurysm and bilateral iliac aneurysm in 1 that made femoral artery cannulation impossible. Five patients had obstruction at the level of the aortic bifurcation (Table 1). Isolated CABG was done in 5 of the 7 patients, CABG and valve repair in 1, and CABG and left ventricular endoaneurysmorrhaphy in 1 (Table 2).

### 2.2. Surgery and Cardiopulmonary Bypass

CPB, surgical techniques, and perioperative management were standardized. Through a median sternotomy, CPB was instituted through cannulation of the ascending aorta and the right atrium with a two-stage venous cannula. The components of the CPB system included a roller pump, a nonheparinized coated circuit, and a hollow fiber membrane oxygenator. Myocardial protection was achieved initially with antegrade St Thomas' crystalloid cardioplegia II after application of the cross-clamp and then continued, with antegrade cold blood cardioplegia being administered into the aortic root after each distal anastomosis and topical cooling. The surgical procedure was performed on moderately hypothermic CPB. Nonpulsatile CPB provided a mean systemic flow of 2.4 L/m^2^ body surface area at mean arterial pressures between 50 and 70 mm Hg. During CPB, the hematocrit was maintained between 20% and 25%. Distal and proximal anastomoses were constructed during a single clamp period. The left internal mammary artery was anastomosed to the left anterior descending coronary artery as the last graft in all patients. Just before the cross-clamp was removed, “warm induction” was applied to all patients.

### 2.3. IABP Catheter Insertion Technique

**Figure 1:**
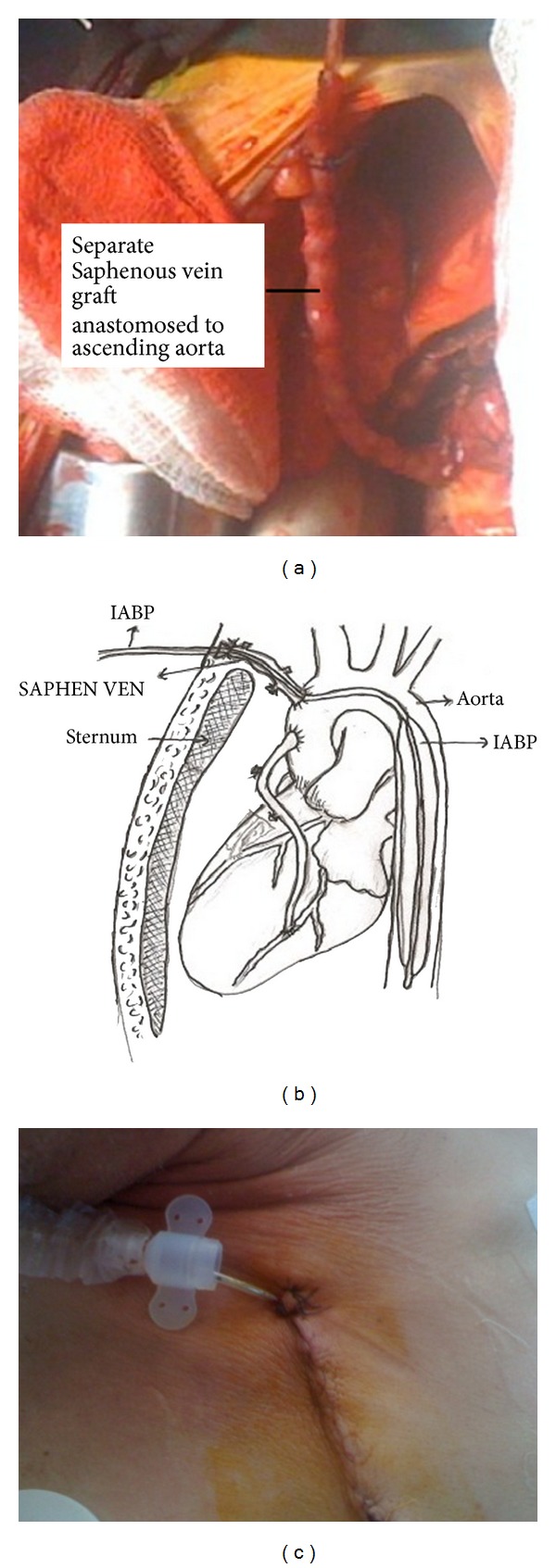
IABP catheter insertion through a separate saphenous vein graft anastomosed to the ascending aorta. (a) Intraoperative picture. (b) Schematic drawing of transaortic IABP procedure. SAPHEN VEN = saphenous vein graft. (c) Appearance of balloon catheter after skin closure.

A separate saphenous vein graft is anastomosed onto an appropriately sized arteriotomy in the ascending aorta with 6-0 polypropylene sutures in an end-to-side fashion under a side-bitting clamp. About a 12 to 15 cm length of the graft is adequate to reach the upper end of sternotomy incision. The bulldog clamp is released and air is removed from the vein graft. The guidewire is advanced selectively through the open end of this graft into the descending thoracic aorta. A 40 cc, 7.5F IABP catheter is then inserted over the gidewire using sheathless technique and the actual inflatable portion of the IABP is positioned to lie in the proximal descending aorta distal to the left subclavian artery and then secured manually with a 2-0 silk ligature to the vein graft (Figures 1(a) and 1(b)). The position of the balloon was readjusted while controlling the descending aorta manually before closing sternum; the saphenous vein graft with the IABP catheter is brought out the upper end of the sternotomy incision (above jugulum) and secured to the skin with a heavy ligature. IABP line is then connected to a Datascope pump (Datascope Corp, Fairfield, NJ). Following improvement of hemodynamic parameters, the patients were successfully weaned off CPB. After sternal closure the vein graft contained the catheter is placed at the upper end of median sternotomy incision. The skin and subcutaneous tissue are approximated over the entire vein graft and IABP with interrupted sutures (Figure 1(c)). The IABP insertion site is covered with sterile dressing. Postoperatively, anticoagulation with heparin sodium is administered intravenously every 4 h of IABP assist according to activated coagulation time.

### 2.4. IABP Removal

**Figure 2:**
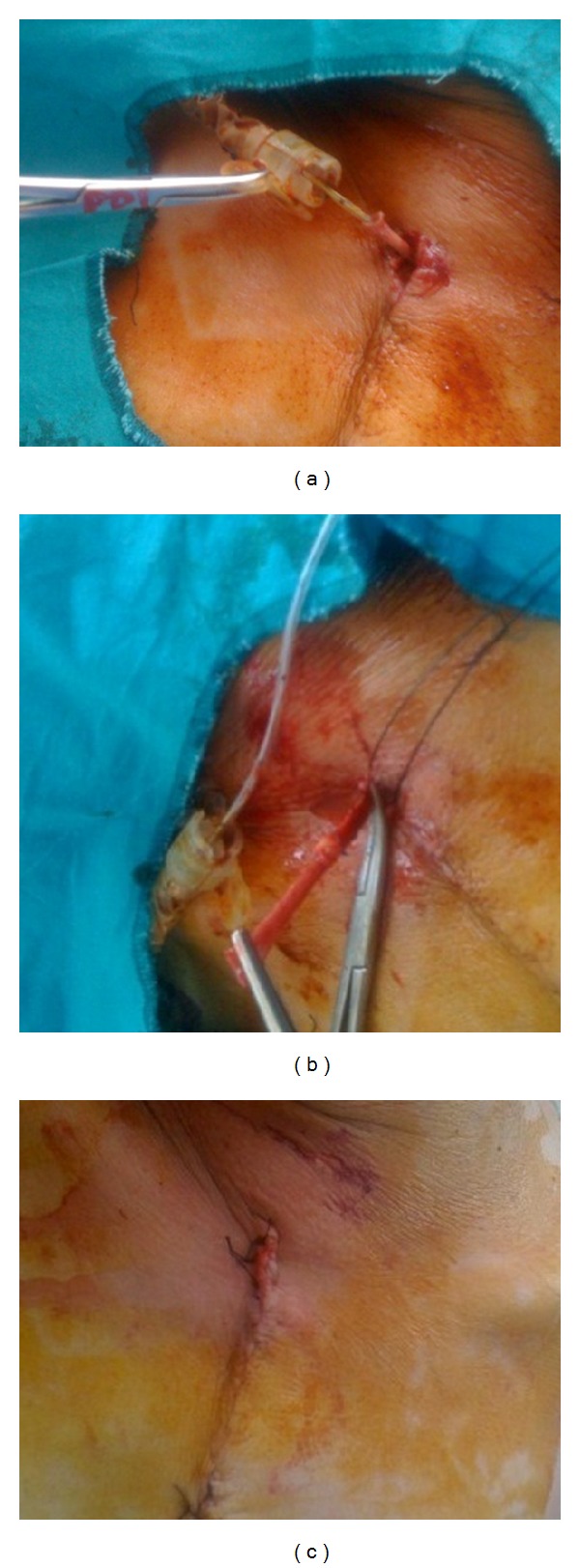
IABP removal and ligation of the graft. (a) The upper sternotomy incision is opened, the securing silk ligature is divided, and IABP catheter is gently removed. (b) A vascular clamp is applied to the graft. The vein graft is ligated at its base close to the sternum and the rest of the graft excised. (c) The graft stump is harbored into the subcutaneous tissue. Skin and the subcutaneous tissue are closed with interrupted sutures.

When hemodynamic stability restored and the need for IABP assistance ended, IABP catheter is easily removed under local anesthesia without resternotomy at the bedside in surgical intensive care unit (ICU). To remove the catheter, the intra-aortic balloon is deflated. The upper portion of sternotomy incision (2 or 3 sutures) is opened, the saphenous vein graft is located, the securing silk ligature is divided, and the IABP catheter is gently removed (Figure 2(a)). A vascular clamp is applied to the graft. The vein graft is ligated at its base close to the sternum and the rest of the graft excised under sterile conditions (Figure 2(b)). The graft stump ultimately is harbored into the subcutaneous tissue. After ample irrigation with dilute povidone-iodine solution, skin and the subcutaneous tissue are closed with interrupted sutures (Figure 2(c)).

### 2.5. Statistical Analysis

Statistical analyses were performed using the SPSS software package (SPSS for Windows, version 19; SPSS Inc, Chicago, IL, USA). Data are presented as the mean ± the standard deviation of the mean or number (percent) when necessary.

## 3. Results

Among the 7 patients receiving transaortic IABP support, 5 (71.4%) were men and 2 (28.6%) were females. Their age ranged from 59 to 76 years (mean, 69.0 ± 5.6 years).

All patients required intraoperative IABP insertion due to difficult weaning from CPB because of intraoperative low cardiac output syndrome. The procedure was performed successfully and the balloon easily inserted in all the patients. Chest roentgenograms were used postoperatively and then daily thereafter to confirm the exact position of the IABP catheter. There were no balloon migrations. All patients had perioperative inotropic support. Four patients received levosimendan additionally.

IABP assistance was maintained in 1 : 1 ratio. The mean duration of IABP support was 54.0 ± 13.4 hours, ranging from 36 to 72 hours. Mean ICU stay was 6.4 ± 2.3 days (range, 3 to 10 days). The postoperative data are given in Table 2.

There were no in-hospital deaths. There were no cases of neurological complications, dissections, bleeding, distal embolizations, or infections attributable to ascending aortic insertion of the IABP catheter. All patients weaned successfully from IABP support. In all the patients, IABP catheters were removed in surgical ICU and no problems were experienced in the early period due to removal of them. In our series, IABP removal did not require repeat sternotomy.

**Figure 3:**
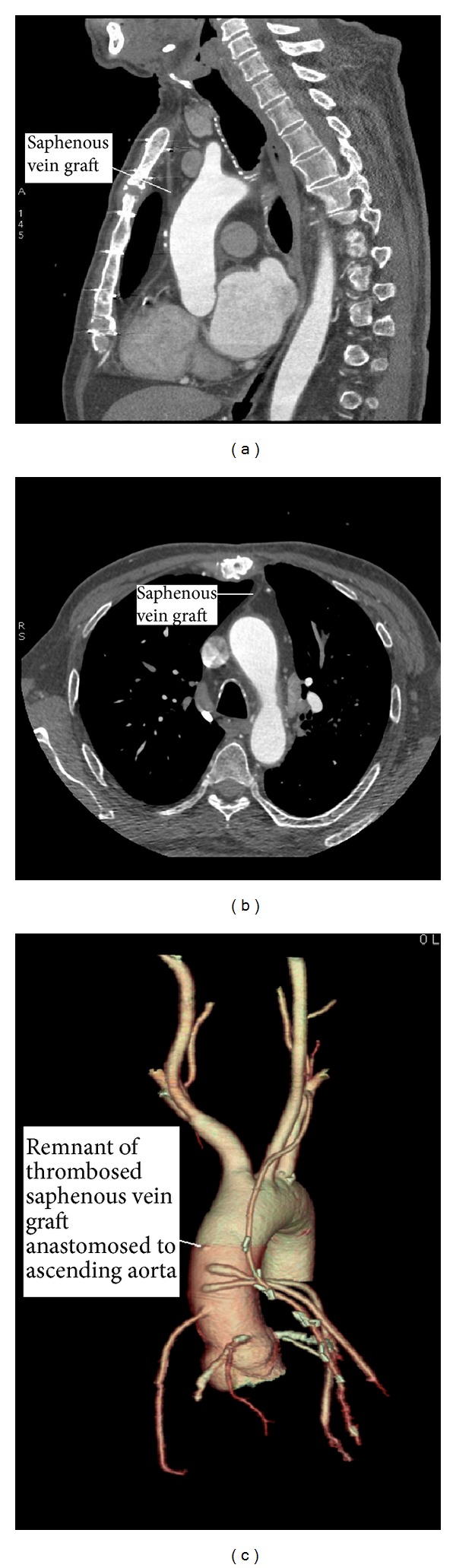
At postoperative 6th month, multislice CT imaging shows thrombotic occlusion at the remnant of the saphenous vein graft. (a) Sagittal section imaging of thrombosed saphenous vein graft. (b) Cross-sectional appearance of thrombosed saphenous vein graft. (c) Three-dimensional display of remnant of thrombosed saphenous vein graft anastomosed aorta.

All patients were discharged home in stable conditions. During the follow-up, all the patients except two were found to be free of symptoms and none died during the follow-up. At postoperative 6th month, multislice CT angiography examination showed thrombotic occlusion at the remnant of the saphenous vein graft (Figures 3(a)–3(c)). However, all coronary artery bypass grafts were patent. No other pathology was found.

## 4. Discussion

IABP is currently the most widely used mechanical circulatory support in cardiac surgical patients during the preoperative and perioperative period [1, 2]. IABP provides haemodynamic stability by assisting myocardial oxygen supply and demand balance, preoperatively, intraoperatively, and during the critical postoperative period. Intraoperative IABP support is needed for patients who cannot be weaned from CPB. The route choice for IABP catheter insertion during CPB is related to accessibility [6]. Conventionally, IABP catheter is usually positioned in the descending aorta through retrograde femoral catheterization. However, in patients with severe aortoiliac occlusive or aneurysmal disease, or small peripheral arteries, femoral route is not possible. In these circumstances, there are several alternative methods to provide counterpulsation. Alternative routes for IABP catheter insertion include the subclavian, axillary, brachial, innominate, or iliac arteries [11–14, 18–23]. Availability of small IABP catheters can broaden the indication for these methods of insertion in an increasing number of patients encountered in daily cardiovascular practice [14]. The catheter can also be inserted intraoperatively using a transaortic route including the ascending aorta or the proximal portion of the aortic arch [10, 24]. Of these alternate approaches, transaortic insertion is the most frequently used and constitutes a rate of 1.9% to 6.2% of all IABP procedures [1] (Table 3).

Our experience seems to confirm that transaortic route is a suitable alternative way to allow IABP insertion in patients with severe aortoiliac diseases. We did not encounter a problem or complication related to this procedure used in failure to wean from CPB. Transaortic route is a good second choice (class I level, C evidence) for intraoperative placement of an IABP in patients with severe aortoiliac disease or prior abdominal aortic or femoral artery operation [1, 5, 7, 24]. In this option, the IABP catheter may be inserted directly into the ascending aorta [7, 16–18, 25, 26] or indirectly through a graft anastomosed to the ascending aorta and brought into the subcutaneous tissues in the jugulum or xiphoid region [5, 6, 8, 9, 25].

The techniques of transaortic IABP insertion have evolved over the past four decades. There is no available ideal technique. This technique should permit a rapid and safe IABP insertion combined easily with its removal and minimal or no residual synthetic material within the mediastinum [9]. In patients needed IABP support during cardiac surgery, an open sternum facilitates direct insertion into the ascending aorta with the balloon catheter tip lying distally in the descending aorta [24]. Direct catheter insertion includes a technique that used pledgeted or concentric pursestring sutures to secure the balloon catheter in the ascending aorta [7, 27]. This graftless technique offers the advantage of rapid balloon placement through the ascending aorta under direct vision. However, it has the disadvantage of requiring a repeat sternotomy for IABP catheter removal. Additionally, there is always the possibility that a thrombus on the balloon catheter might be stripped off by the aortic wall during its removal.

A variety of techniques for inserting the IABP through a graft sutured to the ascending aorta have been reported [5, 6, 8, 9, 15]. These techniques can eliminate the necessity of resternotomy, and the IABP catheter is removed in the surgical ICU under local anesthesia. The use of a graft may help prevent frictional resistance during balloon removal. A technique for insertion of an IABP catheter indirectly into the aorta was described by McGeehin et al. [5], in which a polytetrafluoroethylene vascular graft of 10 mm in diameter is anastomosed to the ascending aorta under a partial occlusion clamp and tunneled behind the sternum below the xiphoid process. Other authors also reported similar techniques [6, 8, 9, 15]. Burack and associates [9] described a technique for transaortic IABP insertion that can be performed in a rapid and atraumatic fashion in 14 patients. In their method, they used a short (4 cm) Gore-Tex vascular graft of 6 mm in diameter and performed the anastomosis without a side-biting vascular clamp by using partial-thickness bites on the aortograft suture line and the synthetic graft was brought out through the sternotomy incision. They also removed IABP catheter without the need to resternotomy.

The technique that Soo and Parissis [15] described is an alternative that obviates the need for resternotomy to remove the IABP catheter. Their technique is unique in that video-assisted thoracoscopic surgery is used, obviating the need to tunnel the IABP through the sternum in the above-described technique by Burack and associates. This method can potentially reduce the incidence of sternal instability and sternal wound infection. In our study, we used indirectly a separate saphenous vein graft anastomosed to the ascending aorta for balloon insertion to provide IABP support in patients who have difficulties for weaning from CPB. In the technique we described, there is no need to return the patient to the operating room. The simple suture set is enough at the bedside in surgical ICU. In all of our patients, the removal of IABP catheter did not require repeat sternotomy.

There are reasons for lack of space in the ascending aorta including very short aortas, anastomoses of multiple saphenous vein grafts, the aortotomy suture lines for aortic valve surgery, or the aortic perfusion or de-airing cannulas [6–10, 27]. These reasons can make the ascending aorta an unsuitable route. In these situations, it is impossible to apply the side-biting clamp to the aorta for additional Synthetic graft implantation because of the absence of free space. In these settings, Nunez and coworkers [8] described a technique in which a woven Dacron graft of 12 mm in diameter is sewn to the ascending aorta without the use of a partial occlusion clamp using partial-thickness sutures in the aortic wall. In our study, we could easily find sufficient anastomotic area for a separate saphenous vein graft anastomosis by applying the partial occlusion clamp even when the aorta is crowded with multiple saphenous vein grafts. With this alternative method, we observed that there is no increased risk of mediastinal contamination and sternal wound infection.

The base of the balloon should lie approximately 2 cm below the left subclavian artery. Santini and Mazzucco [16] recommended a simple technique to achieve correct transaortic IABP catheter insertion and positioning without the need for special equipment. The external pressure is applied to the left subclavian artery to avoid displacement of catheter. Thereafter, balloon position is guided by means of palpation into descending thoracic aorta through the opened pleura. Thus, by this manipulation aberrant cannulation to the cerebral arteries or left subclavian artery is prevented during the insertion of the catheter. In our study, we also made a similar application. Transesophageal echocardiography is often used to guide appropriate IABP positioning in the descending thoracic aorta in the operating room. As well, epiaortic ultrasound can be used to confirm the position of the catheter [6, 9, 17, 18, 23].

Transaortic IABP insertion is associated with considerable morbidity and mortality. Possible complications related to this route include aortic dissection, bleeding at the anastomosis or directly aortic insertion site, cerebral or peripheral embolism, myocardial infarction, mediastinal or graft infection, balloon rupture, aberrant cannulation of the subclavian artery, or improper positioning [5–7, 9, 27]. These problems can be minimized by careful surgical techniques. Transaortic IABP insertion should be avoided in patients with aortic dissection, a severe calcified ascending aorta, or obvious ultrasonographic evidence showing potential embolic debris in the ascending aorta.

McGeehin et al. [5] reviewed 39 patients who required transthoracic IABP insertion. Five patients (13%) sustained complications potentially related to the procedure including balloon rupture in 2 patients (5%), graft infection in 1 (2.5%), and cerebrovascular accidents in 4 (10%). The overall survival was 44% (17/39). There were no deaths directly related to the balloon placement or removal. In Meldrum-Hanna and colleagues' [6] series of 8 patients, they encountered the complications related to transaortic IABP including graft infection, aberrant cannulation of the left subclavian artery, left coronary artery embolism, and inability to close the sternum due to mechanical tamponade. In our study, there were no complications and mortality related to transaortic IABP.

In a retrospective large series of 100 cases of transthoracic IABP insertion without graft, Hazelrigg and coworkers [7] evaluated the complications in 81 patients who survived to have their IABP removed. They demonstrated no increased mortality and complication rates similar to those of femoral insertion. The authors also reported that complications related to transaortic route included balloon rupture in 5 patients (6.2%), cerebral vascular accident in 2 (2.5%), transient ischemic attack in 1 (1.2%), bleeding at the IABP arteriotomy site in 3 (3.7%), and mediastinal infection in 3 (3.7%). In this high risk group of patients, the rate of balloon rupture and mediastinal bleeding and infection has increased because of the direct transaortic IABP catheter insertion. However, neurologic events do not appear to be increased. In their series, overall mortality was 27% [7]. Santini and associates [28] also described balloon migration as an unexpected complication of IABP support via the ascending aorta. They explained that possible reason for this complication may be the looping of the balloon catheter into the aortic lumen possibly prompted by a too proximal location.

Pinkard and associates [29] found in analyzing a series of 123 IABPs inserted for weaning from CBP in CABG patients (42 transaortic and 81 femoral) that the increased mortality in the arch insertion group was a result of the greater comorbidities rather than the route of insertion. Additionally, they remarked no increase in complications in the aortic insertion group, in spite of a higher incidence of leg complications in the patients with femoral insertion.

In our experience, the transaortic route appears as an excellent intraoperative solution to provide IABP support in patients who are difficult to wean from CPB due to low cardiac output syndrome and who are unsuitable femoral access. We did not observe any problems or complications related to the placement or use of the transaortic IABP. An important advantage of our technique is technically easier and safer to sew the saphenous vein graft to the ascending aorta compared with the synthetic vascular grafts. An another advantage is that the separate saphenous vein graft which anastomosed the ascending aorta runs for a short distance behind the manubrium and does not pass through or behind the sternotomy, as described by other authors. These benefits can result in a decreased risk of sternal wound infections. Furthermore, the use of a sheathless balloon inserted through the saphenous vein graft makes balloon removal easy and safe at the bedside under local anesthesia.

*Limitation of the Study*. Herein, we reported our institutional experience with small case series, even if larger studies are needed to definitely verify the advantages of transaortic route. This route to provide IABP assistance is lifesaving in patients with severe comorbidity in whom transfemoral route failed or contraindicated. Transaortic IABP insertion has the following drawbacks: (a) it requires the environment of operating room; (b) it is more time consuming than the femoral route; and (c) it has several potential serious complications, including the risk of cerebral emboli, aortic dissection, and haemorrhage from the graft sutured to ascending aorta during catheter removal because of overstretch.

## 5. Conclusions

IABP catheter insertion through a separate saphenous vein graft anastomosed to the ascending aorta for IABP counterpulsation is a simple, reliable, and reproducible option in patients with severe aortoiliac disease in whom retrograde femoral route is not possible. Although possible severe complications are associated with ascending aortic cannulation, transaortic route should be considered for all patients requiring intra- and postoperative IABP support in whom other access routes are not feasible. We also recommend that this access site should be added to the surgical armamentarium in these complicated pathologies.

## Figures and Tables

**Table 1:** The preoperative clinical details of the patients.

Patient number	Age (years)/gender	Preoperative LVEF (%)	Risk factors	Aortoiliac vascular lesions
1	59/M	28	HT, hypothyroidism	90% stenosis, bifurcation of the abdominal aorta
2	67/F	30	HT, DM, COPD, morbid obesity	90% stenosis, right common iliac artery; occluded left common iliac artery
3	71/M	30	HT, DM	Occluded distal abdominal aorta
4	69/M	25	HT, DM, CRD	Occluded right internal iliac artery; occluded left common iliac artery
5	76/F	28	HT, DM, obesity	80% stenosis, right common iliac artery; 90% stenosis, left internal iliac artery; occluded right femoral artery
6	67/M	27	HT, DM	occluded left internal iliac artery; 90% stenosis, right common femoral artery
7	74/M	30	HT, COPD	Bilateral proximal iliac arterial aneurysms; 80% stenosis, left common femoral artery

COPD: chronic obstructive pulmonary disease; CRD: chronic renal disease; DM: diabetes mellitus; HT: hypertension.

**Table 2:** Operative and postoperative data of the patients.

Patient number	Operation performed	Inotropic support	Duration of IABP support (hours)	IABP removal period (minutes)	Intensive care unit stay (days)
1	CABG × 3	Dopamine; dobutamine; levosimendan	36	7	2
2	CABG × 4	Dopamine; dobutamine; norepinephrine	48	12	5
3	CABG × 3 + mitral ring annuloplasty	Dopamine; dobutamine; norepinephrine	52	10	7
4	CABG × 4	Dopamine; dobutamine; norepinephrine; levosimendan	72	9	8
5	CABG × 4	Dopamine; dobutamine; levosimendan	46	12	5
6	CABG × 3 + left ventricular endoaneurysmorrhaphy	Dopamine; dobutamine; norepinephrine	72	8	7
7	CABG × 3	Dopamine; dobutamine; norepinephrine; levosimendan	52	15	10

CABG: coronary artery bypass grafting; IABP: intra-aortic balloon pump.

**Table 3:** A series of transaortic IABP catheter insertion.

Reference Year	No. of the patients	Balloon insertion	Balloon removal	Mean duration of IABP support (hours)	Complications	Outcome
McGeehin et al. [5] 1987	39	Indirectly, synthetic graft	CCD (19) OCD (2)	83	Balloon rupture (2), transient ischemic attack (1), graft infection (1), CVA (4)	Overall survival 44%
Meldrum-Hanna et al. [6] 1985	8	Indirectly, synthetic graft	CCD (4) OCD (4)	48	Graft infection, aberrant cannulation of left subclavian artery, left coronary embolism, inability to close sternum	Five patients alive
Hazelrigg et al. [7] 1992	100	Directly	OCD	40.7	Balloon rupture (5), CVA (2), bleeding at arteriotomy site (1), mediastinal infection (3)	Overall mortality 27%
Nunez et al. [8] 1980	3	Indirectly, synthetic graft	N/A	48	None	One patient alive
Burack et al. [9] 1996	14	Indirectly, synthetic graft	CCD	52.8	Balloon rupture (2), peripheral and cerebral emboli (1), minor wound infection (2)	57% of the patients were discharged
Present study	7	Indirectly, saphenous vein graft	CCD	54	None	All patients alive

Brackets show the number of patients. References of a case report were not included in Table 3.

IABP: intra-aortic balloon pump; OCD: open chest decannulation (resternotomy); CCD: closed chest decannulation (no resternotomy); CVA: cerebrovascular accident; N/A: not available.
